# Effects of myocardial fibrosis and ventricular dyssynchrony on response to therapy in new-presentation idiopathic dilated cardiomyopathy: insights from cardiovascular magnetic resonance and echocardiography

**DOI:** 10.1186/1532-429X-13-S1-P338

**Published:** 2011-02-02

**Authors:** Darryl P Leong, Adhiraj Chakrabarty, Nicholas Shipp, Payman Molaee, Per Lav Madsen, Lucas Joerg, Thomas Sullivan, Hugh Greville, Stephen G Worthley, Carmine G De Pasquale, Prashanthan Sanders, Joseph B Selvanayagam

**Affiliations:** 1 Department of Cardiovascular Medicine, Flinders Medical Centre, Adelaide, Australia, The University of Adelaide, Adelaide, Australia, The Flinders University of South Australia, Adelaide, Australia; 2 Department of Cardiovascular Medicine, Flinders Medical Centre, Adelaide, Australia, The Flinders University of South Australia, Adelaide, Australia; 3 University of Adelaide, Adelaide, Australia, Adelaide, Australia; 4 Department of Cardiovascular Medicine, Flinders Medical Centre, Adelaide, Australia, Adelaide, Australia; 5 Discipline of Public Health, University of Adelaide, Adelaide, Australia; 6 Department of Respiratory Medicine, Royal Adelaide Hospital, Adelaide, Australia

## Objectives

To determine whether the extent of myocardial fibrosis by late-gadolinium enhancement cardiovascular magnetic resonance (LGE-CMR), and echocardiographic ventricular dyssynchrony can independently predict response to medical therapy in patients with newly-diagnosed idiopathic dilated cardiomyopathy (DCM).

## Background

Myocardial fibrosis and ventricular dyssynchrony are frequent findings in DCM. Although previous studies have reported poor prognosis with the presence of myocardial fibrosis in DCM, they focussed on patients with established cardiomyopathy, and did not characterise patients early in the disease course and may not have included those with significant improvement in LV function soon after diagnosis. Hence, the degree of myocardial fibrosis and ventricular dyssynchrony at initial presentation, and their role in perpetuating left ventricular (LV) dysfunction in DCM remains unclear. We hypothesised that extent of myocardial fibrosis by the LGE-CMR technique and echocardiographic ventricular dyssynchrony are independent predictors of failure of improvement in LV function in new-onset DCM.

## Methods

Patients with a new diagnosis of DCM (LV ejection fraction (EF) ≤45%) made within the preceding two weeks were recruited. Patients underwent LGE-CMR, echocardiography, 6-minute-walk testing, cardiopulmonary exercise testing, and blood sampling for measurement of serum NT-pro-BNP concentration at baseline. Baseline patient characteristics were compared with a cohort of healthy volunteers. Myocardial fibrosis by LGE-CMR was identified by experienced observers blinded to patient outcome, and was quantified by planimetry for calculation of fibrosis mass. LV systolic function was reassessed after 5 months optimal medical therapy.

## Results

Sixty-eight patients with DCM and 19 healthy volunteers were studied. DCM patients were studied a median 12.5 days following diagnosis. Compared to healthy controls, DCM patients exhibited poorer functional capacity, higher serum NT-pro-BNP concentration, and greater inter- and intraventricular dyssynchrony. Twenty-four percent of DCM patients exhibited LGE at diagnosis, whereas no LGE was observed amongst controls. Within DCM patients with LGE, the mean fibrosis mass was 2.2±1.3g. On multivariate analysis, strain dyssynchrony index, and fibrosis mass were independent predictors of change in LVEF over time (p≤0.001) (Table 1). LGE-CMR conferred additive prognostic value over and above clinical and echo-dyssynchrony parameters for the prediction of improvement in LVEF.

**Table 1:** Uni- and Multivariate Model of Prognostic Factors for Change in LVEF

Variable	Univariate model		Multivariate model	
	Coefficient (95% CI)	p-value	Coefficient (95% CI)	p-value

Age	-0.06 (-0.3 to 0.2)	0.8		
Gender	-4 (-10 to 3)	0.8		
6-minute walk distance (m)	0.03 (-0.01 to 0.08)	0.3		
VO_2 PEAK_ (mL/kg/min)	0.3 (-0.7 to 1.3)	0.6		
Tissue Doppler dyssynchrony index (ms)	-0.07 (-0.3 to 0.2)	0.7		
E/E’	0.04 (-0.2 to 0.3)	0.8		
Left atrial volume (mL)	-0.02 (-0.1 to 0.1)	0.8		
TAPSE (cm)	-1 (-10 to 7)	0.7		
Interventricular mechanical delay (ms)	-0.1 (-0.3 to 0.05)	0.3	0.3 (-0.06 to 0.6)	0.1
NT-pro BNP (μg/L)	-1 (-2 to -0.09)	0.007	-0.2 (-2 to 1)	0.8
QRS-duration (ms)	-0.14 (-0.3 to -0.02)	0.004	-0.3 (-0.7 to 0.1)	0.2
Strain dyssynchrony index (ms)	-0.1 (-0.2 to -0.06)	0.04	-0.1 (-0.2 to -0.04)	<0.001
Fibrosis mass (g)	-7 (-11 to -3)	<0.001	-7 (-11 to -4)	<0.001

## Conclusions

The extent of myocardial fibrosis is a powerful predictor of a negative response to medical therapy in new-presentation DCM, and LGE-CMR may thus be an important risk-stratifying investigation in these patients. Accurate risk stratification may permit more targeted pharmacological and device therapies for patients with newly-diagnosed DCM.

